# Arcyriaflavin A Alleviates Osteoporosis by Suppressing RANKL-Induced Osteoclastogenesis

**DOI:** 10.3390/ijms26052141

**Published:** 2025-02-27

**Authors:** Mengbo Zhu, Mingwei Xu, Damien Bertheloot, Victoria C. Brom, Alexander Sieberath, Jochen Salber, Kristian Welle, Christof Burger, Dieter C. Wirtz, Shaowei Wang, Frank A. Schildberg

**Affiliations:** 1 Department of Orthopedics and Trauma Surgery, University Hospital Bonn, 53127 Bonn, Germany; 2 Department of Orthopedics, The Second Hospital of Shanxi Medical University, Taiyuan 030013, Chinawangshaowei@sxmu.edu.cn (S.W.); 3 Department of Experimental Surgery, Centre for Clinical Research, Ruhr-Universität Bochum, 44780 Bochum, Germany; 4 Department of Surgery, Universitätsklinikum Knappschaftskrankenhaus Bochum GmbH, 44892 Bochum, Germany

**Keywords:** osteoclasts, osteoporosis, Arcyriaflavin A, osteoimmunology, musculoskeletal immunology

## Abstract

Osteoclasts (OCs) are important therapeutic targets in the treatment of osteoporosis. The aim of this study was to explore a novel therapeutic approach for osteoporosis using Arcyriaflavin A (ArcyA), a natural compound derived from the marine invertebrate *Eudistoma* sp. We systematically evaluated the effects of ArcyA on OC differentiation and function in mouse models using molecular biology assays, cellular function analyses and in vivo animal experiments. We also evaluated the efficacy of ArcyA in human cells. The TRAP staining results provide the first clear evidence of the drug’s inhibitory effect, whereby the administration of ArcyA led to a significant reduction in TRAP-positive cells compared to the control group at concentrations that were non-toxic to bone marrow macrophages. Meanwhile, a significant reduction in the number of multinucleated giant cells with more than ten nuclei was observed. Furthermore, similar TRAP staining results were reproduced in human OCs, suggesting that ArcyA has the same effect on OCs derived from human PBMCs. At the molecular level, ArcyA treatment resulted in the downregulation of genes relevant to OC differentiation (NFATc1, cFos and TNFrsf11α), fusion and survival (DCstamp and ATP6v0d2) and resorption function (CTSK, MMP9, integrin β3 and ACP5). A western blot analysis of the corresponding proteins (NFATc1, cFos, CTSK and integrin β3) further confirmed the PCR results. Furthermore, ArcyA-treated OCs produced significantly fewer resorption pits, indicating suppressed bone resorption activity. Consistent with this, in vivo experiments using an ovariectomy (OVX)-induced osteoporosis mouse model showed that ArcyA treatment significantly alleviated bone loss. Mice in the treatment groups had higher BV/TV values, and this therapeutic effect was enhanced in a dose-dependent manner. In addition, our research also showed that IκB could be a potential target for the inhibitory effect of ArcyA. In conclusion, these findings suggest that ArcyA has significant therapeutic potential for the treatment of osteoporosis by inhibiting osteoclastogenesis and bone resorption. Further studies are warranted to explore its clinical applications.

## 1. Introduction

Population ageing is a significant global issue. It is estimated that the population over 60 will double, and those over 80 will quadruple, within the next three decades [1]. Osteoporosis is an age-related disease characterized by deteriorated bone tissue, reduced mineral density, and low resistance to impact [2]. Studies have shown that hip fractures resulting from osteoporosis plague around one-third of females and one-sixth of males in the population aged over 65 years [3]. A retrospective observational study of over 10 million osteoporosis patients for the entire year of 2018 indicated that more than 5 million are considered to be at high risk of bone fractures, accounting for 48.7% [4]. This indicates that osteoporosis, along with the secondary fractures it causes, undermines patient’s quality of life (QOL), and imposes an increasing financial burden on healthcare systems annually [5]. Therefore, developing effective, affordable, and accessible preventive and therapeutic strategies against osteoporosis is a necessary breakthrough to achieve to address the growing challenges posed by population ageing.

In clinical practice, conservative medical therapies are primarily used to treat severe osteoporosis caused by various etiologies, including PMO (postmenopausal osteoporosis) and cancer-associated osteoporosis [6]. The clinically common drugs include, but are not limited to, bisphosphonates, estrogen-related therapy, denosumab, and parathyroid hormone analogues [7]. As a first-line therapy, bisphosphonates can lead to osteonecrosis of the jaw and atypical subtrochanteric and diaphyseal femoral fractures, as well as inherent stress on the stomach, liver, and kidneys [8,9]. Consequently, this limits its long-term use. Even though the concept of a “drug holiday” has been proposed to reduce the side effects of bisphosphonates, this adds to the complexity of clinical and nursing practice, and requires substitute medications [10]. As an alternative drug to bisphosphonates, denosumab is not perfect in clinical applications because of the potential multiple spontaneous vertebral fractures caused by discontinuation [11], and its high cost. Therefore, the active exploration of OC inhibitors may help to introduce new ideas for clinical alternative or combination therapies. Under physiological conditions, osteoclasts (OCs) act as a critical player in bone resorption and remodeling by creating an acidic microenvironment and secreting proteolytic enzymes, such as Cathepsin K (CTSK) and matrix metalloproteinases (MMP) [12,13]. However, under pathological conditions, OCs mediate excessive bone loss and structure damage [14], which makes them a key therapeutic target in osteoporosis treatment.

Rather than focusing on classical cell signaling pathways, we were directed towards the bone microenvironment. Macrophage polarization influences the activation and differentiation of OCs [15]. M2 cytokines suppress the differentiation of OC precursors and inhibit bone resorption [16]. In contrast, M1 macrophages serve as the precursor of OC and secrete osteoclastogenesis-promoting cytokines [17,18]. Along these lines, we noted that a previously published study, which investigated differentially expressed genes using RNAseq, highlighted several M2-activating compounds, including Arcyriaflavin A (ArcyA) [19]. This underreported compound, which was originally isolated from the marine invertebrate *Eudistoma* sp., has been documented for its inhibition of cyclin-dependent kinase 4 (CDK4) and anti-tumor effects [20]. As a drug that induces an M2 phenotype, we were wondering if it would also inhibit OC differentiation and function.

Our study is the first to demonstrate that synthetic ArcyA suppresses osteoclastogenesis and alleviates bone loss both in vitro and in vivo. Experiments performed on mouse bone marrow-derived macrophages (BMMs) provide evidence from aspects of cytological analysis, as well as gene and protein expression. Moreover, our in vitro findings are corroborated by results from an in vivo mouse model and human-derived primary cells.

## 2. Results

### 2.1. ArcyA Suppresses Osteoclastogenesis Induced by RANKL

**Figure 1:**
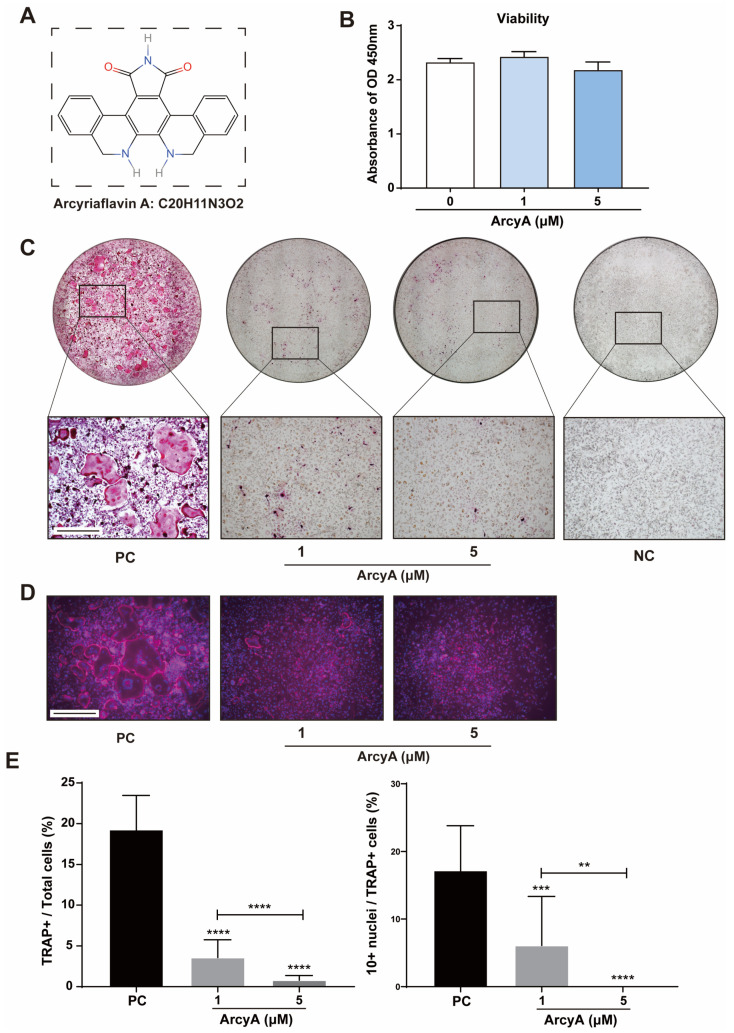
(**A**) The molecular structure and formula of Arcyriaflavin A (ArcyA). The molecular structure was generated using the InDraw software V6 (https://www.integle.com/static/indraw; accessed on 23 February 2025). (**B**) The cytotoxicity of ArcyA assessed through a CCK-8 assay based on cell viability. (**C**) TRAP staining of groups treated with or without ArcyA at different concentrations (1 μM and 5 μM). Non-RANKL-stimulated cells were included as negative control (NC). PC represents the positive control. Scale bar = 500 μm. (**D**) Fluorescent staining of ArcyA-treated cells. Cytoskeleton with magenta fluorescence indicates actin staining and dots with blue fluorescence indicate DAPI staining. Scale bar = 500 μm. (**E**) Quantitative analysis of ratio of TRAP-positive cells (≥3 nuclei, red stained) among all included cells in 0.01 cm^2^, as well as the ratio of >10 nuclei cells among all TRAP-positive cells. N = 3. Data are presented as mean ± SD, with statistical significance indicated ** *p* ≤ 0.01, *** *p* ≤ 0.001, **** *p* ≤ 0.0001.

To ensure that ArcyA (Figure 1A) had no toxic effects on osteoclastogenesis, we evaluated the influence of ArcyA on BMMs’ viability, the precursor of OCs. CCK8 testing showed no significant differences in cellular viability between the treatment groups and the control group (Figure 1B), indicating that ArcyA did not affect BMM viability.

TRAP staining was performed to assess the inhibitory effect of ArcyA on OC differentiation. These results demonstrate that ArcyA inhibited the differentiation of BMMs into OCs. In Figure 1C, we see that the positive control showed the highest multinuclear red-stained cells, identified as TRAP-positive cells. TRAP-positive cells were present in the ArcyA treatment groups (1 μM and 5 μM) compared to the negative control (NC) group, but numbered significantly fewer than those in the positive control (PC) group. Additionally, fluorescent staining was used to visualize the differentiation of multinucleated giant cells in the different groups. In the presence of ArcyA, the number of giant cells with more than 10 nuclei in both the 1 and 5 μmol groups was lower than that in the positive control group (Figure 1D). Concurrently, quantitative data support the microscopy results, showing that the intervention reduced the formation of OCs and the proportion of cells with more than 10 nuclei. A comparison between the two treatment groups indicates that the higher dose had a more pronounced inhibitory effect, as evidenced by fewer TRAP-positive cells in the 5 μM group than in the 1 μM group. Furthermore, the ratio of cells with more than 10 nuclei in the 5 μM group was less than that in the 1 μM group (Figure 1E).

### 2.2. OC Differentiation and Function-Related Genes Were Downregulated by ArcyA

**Figure 2:**
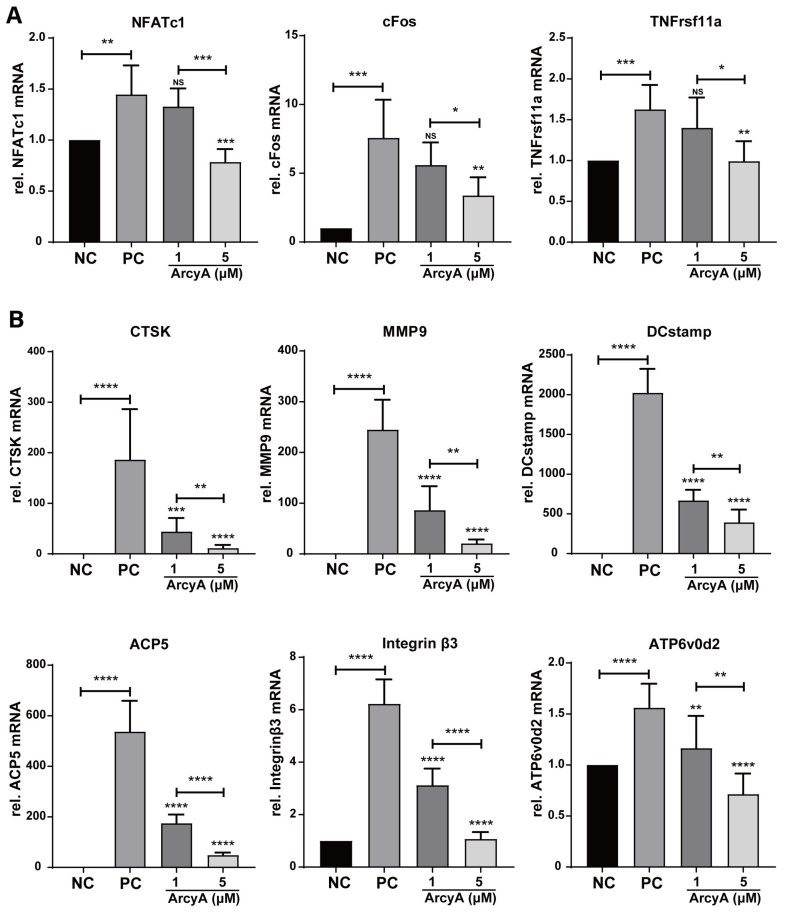
(**A**) Genes related to OC activation, including NFATc1, cFos and TNFrsf11α, were downregulated by ArcyA. NC represents the negative control, PC the positive control. (**B**) Genes related to OC function, including CTSK, MMP9, DCstamp, ACP5, Integrin beta3, and ATP6v0d2, were downregulated by ArcyA. Expression data are relative to Glyceraldehyde-3-phosphate dehydrogenase (GAPDH). Data (N = 5) are presented as mean ± SD, with statistical significance indicated as * *p* ≤ 0.05, ** *p* ≤ 0.01, *** *p* ≤ 0.001, **** *p* ≤ 0.0001, ns indicates no significant difference.

For a comprehensive evaluation, nine genes related to OC differentiation (NFATc1, cFos, and TNFrs11a) and function (CTSK, MMP9, DCstamp, ACP5, Integrin β3, and ATP6v0d2) were analyzed by qPCR (Figure 2).

All these genes were significantly upregulated in the PC group compared to the NC group in the presence of the stimulating factor receptor activator of nuclear factor kappa-B ligand (RANKL). Regarding the treatment groups, the mRNA levels of OC differentiation-related genes, including NFATc1, cFos, and TNFrsf11a, decreased in the presence of 5 μM ArcyA. In contrast, 1 μM ArcyA did not lead to the significant downregulation of these genes (Figure 2A). Similarly, the downregulation of mRNA levels was observed in downstream genes related to specific cellular behaviors with the intervention of ArcyA. Statistically significant results were obtained in both the 1 μM and 5 μM groups (Figure 2B).

### 2.3. ArcyA Suppresses the Resorptive Function of OCs

**Figure 3:**
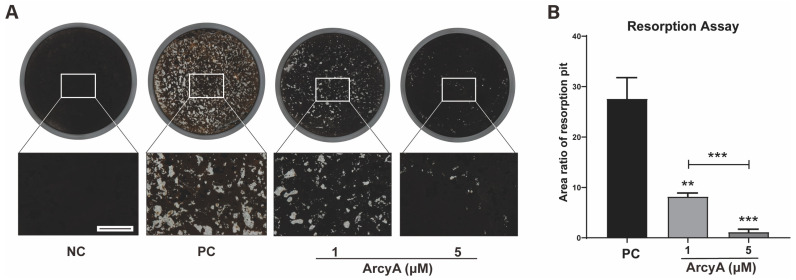
Resorption assay demonstrating the inhibitory effects of ArcyA on OC resorption function. (**A**) NC represents the negative control, where only M-CSF was present in the medium, resulting in no OC formation and no resorption pits. The black coating layer in the NC group shows the original surface appearance. PC represents the positive control. The white areas are resorption pits generated by OCs. Scale bar represents 500 μm. (**B**) Quantitative analysis of average areas of resorption pits in each group. Data (N = 3) are presented as mean ± SD. Statistical significance indicated as ** *p* ≤ 0.01, *** *p* ≤ 0.001.

Bone resorption is a critical function of OCs in the bone microenvironment. Therefore, we conducted a bone resorption assay as a necessary confirmation of the results of molecular mechanism studies. No resorption pits were generated on the coating in NC groups, and thus, these completely dark dyed coatings were defined as 100% (Figure 3A). In the PC groups and experimental groups, functional OCs resorbed the coating, rendering the absorbed areas unstainable. After staining, these areas were transparent, similar to normal 96-well cell culture plates, and appeared white under the microscope. Thus, white areas were defined as resorption pits.

Using ImageJ, the area of the resorption pits was measured and divided by the total area of the coating to calculate the percentage of the coating resorbed by OCs. In Figure 3A, the coating in PC groups was dotted with resorption pits densely. In contrast, the scatter of resorption pits in the 1 μM group was significantly lower than that in the PC group. Resorption pits were sparsely sprinkled in the wells of the 5 μM group.

Quantitative analysis further revealed that the average percentage of the total area of resorption pits was approximately 30% in the PC group, less than 10% in the 1 μM group, and approximately 1% in the 5 μM group. These differences are statistically significant and demonstrate the inhibitory effect of ArcyA on OC resorption activity. Our data also show that higher concentrations of ArcyA had a stronger inhibitory effect.

### 2.4. ArcyA Downregulated the Expression of Proteins Related to OC Differentiation and Function

**Figure 4:**
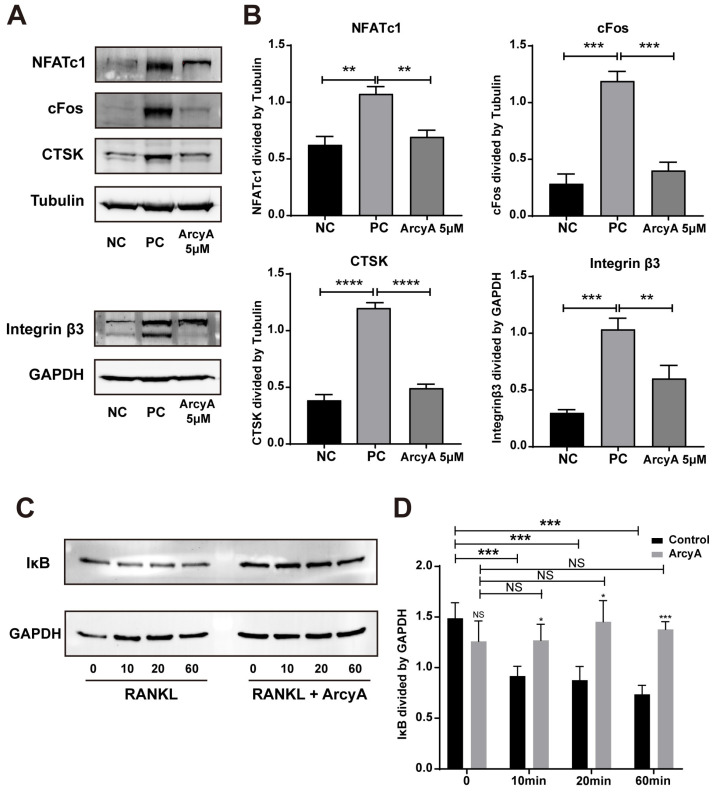
(**A**) Western blots showing the expressions of proteins involved in osteoclastogenesis with and without ArcyA treatment. Tubulin was used as control for NFATc1, cFos and CTSK, while GAPDH served as the control for Integrin β3. (**C**) Western blots showing the expression of IκB, a key factor in the NF-κB signaling pathway, with and without ArcyA treatment. GAPDH was used as the control. The induction times (in minutes) with RANKL or RANKL + ArcyA were as depicted. (**B**,**D**) Quantitative analysis of protein expression levels in each group. Data (N = 3) are presented as mean ± SD. Statistical significance indicated as * *p* ≤ 0.05, ** *p* ≤ 0.01, *** *p* ≤ 0.001, **** *p* ≤ 0.0001, ns indicates no significant difference.

To mutually reinforce our PCR results, differentiation-related proteins (NFATc1 and cFos), function-related proteins (CTSK and Integrin β3), and a key protein factor of the NF-κB signaling pathway (IκB) were analyzed by western blot. In the NC group, the blots of NFATc1 and cFos, two critical upstream protein factors relevant to OC differentiation, were faint. In contract to the NC groups, thicker bands of both NFATc1 and cFos were observed in the PC groups. However, in the 5 μM ArcyA treatment group, the thickness of bands was visually reduced. Explicit quantitative data from the ImageJ analysis demonstrate that the presence of ArcyA significantly downregulated the expressions of both NFATc1 and cFos. Additionally, compared to the NC group, Western blots and quantitative data indicate that CTSK and Integrin β3, both relating to the resorption function, were significantly downregulated when 5 μM of ArcyA was added to the cell cultures, compared to the PC group (Figure 4A,B).

To investigate the influence of ArcyA on early-stage signaling pathways, we tested protein samples collected at various time points within the first hour after RANKL was introduced into the culture system. IκB, a critical inhibitor of the NF-κB pathway, was selected as the target protein and different time points (10, 20, and 60 min) were chosen to monitor the change in IκB expression levels. From the blot images and quantitative analysis, we see no significant difference of IκB level between control and ArcyA-treated group before the introduction of RANKL. However, it can be observed that the level of IκB protein decreased after the addition of RANKL in the control group without ArcyA pre-treatment. In contrast, the expression of IκB did not change significantly in the ArcyA-pre-treated groups. In summary, the expression level of IκB in the ArcyA-treated group was higher than that in the control at all time points, indicating that the presence of ArcyA upregulated the inhibitor during early osteoclastogenesis (Figure 4C,D).

### 2.5. ArcyA Exerts Inhibitory Effect in Human Cells

**Figure 5:**
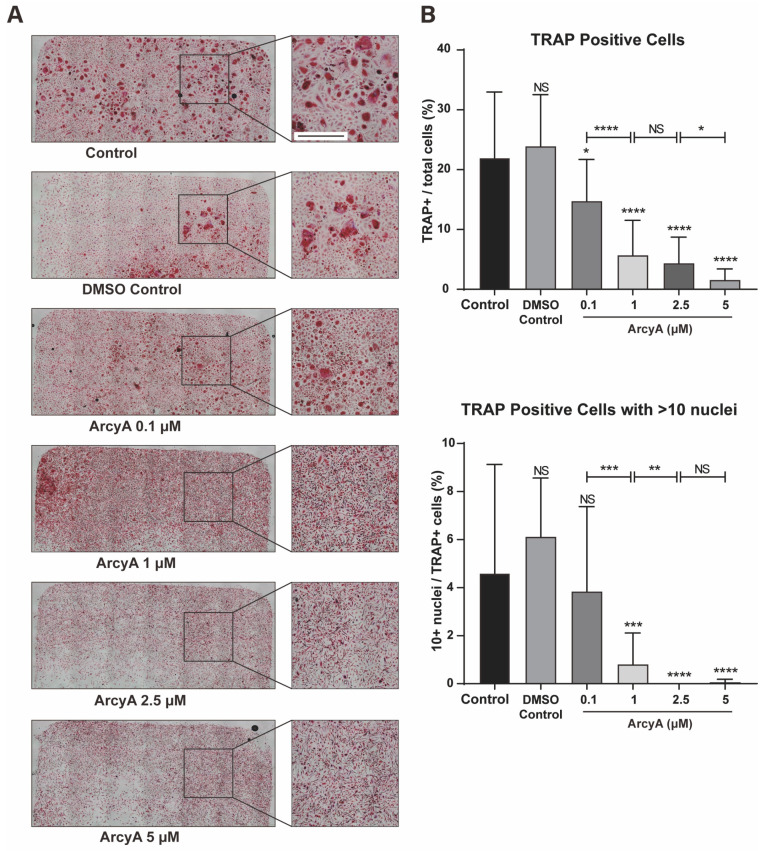
(**A**) TRAP staining of groups treated with ArcyA. Scale bar = 500 μm. (**B**) Quantitative analysis of the ratio of TRAP-positive cells (≥3 nuclei, red stained) among all included cells in a 0.01 cm^2^ sized area. Additionally, the analysis includes the ratio of cells with >10 nuclei among all TRAP-positive cells. Data (N = 4) are presented as mean ± SD, with statistical significance indicated as * *p* ≤ 0.05, ** *p* ≤ 0.01, *** *p* ≤ 0.001, **** *p* ≤ 0.0001, ns indicates no significant difference.

Given the robust results from our murine in vitro experiments, we believe it is essential to test the effects of ArcyA on human-derived OCs. By quantifying the proportion of TRAP-positive cells in the total cell population in different treatment groups, ArcyA’s inhibitory effects on OC differentiation in human cell cultures can be assessed (Figure 5).

There was no significant difference in the number of TRAP-positive cells between 0.1% DMSO and the control group. However, when 0.1 μM of ArcyA was added into system, the number of TRAP-positive cells began to decrease. Compared to the control group, in the group treated with 1 μM, 2.5 μM, and 5 μM, ArcyA showed a more significant reduction in the number of TRAP-positive cell numbers. Moreover, the inhibitory effect of ArcyA demonstrated a gradient-increasing trend. The total number of TRAP-positive cells at 1 μM was significantly lower than that at 0.1 μM, and the 5 μM group was significantly lower than the 2.5 μM group. There was no statistically significant difference between the 2.5 μM and 1 μM groups.

Additionally, we calculated the proportion of giant cells with more than 10 nuclei among TRAP-positive cells in each group. There were no significant differences among the control, the DMSO control, and the 0.1 μM group. However, the ratio of >10 nuclei giant cells was decreased in 1 μM and groups with higher concentrations. Specifically, regarding the proportion of more than 10 nuclei giant cells, that in the group treated with 1 μM of ArcyA was lower than that in the group with 0.1 μM, and that in the group with 2.5 μM was lower than that in the group with 1 μM. Because there were almost no >10 nuclei giant cell in the 2.5 μM and 5 μM groups, there was no statistical difference between these two groups.

### 2.6. ArcyA Alleviated Bone Loss In Vivo

**Figure 6:**
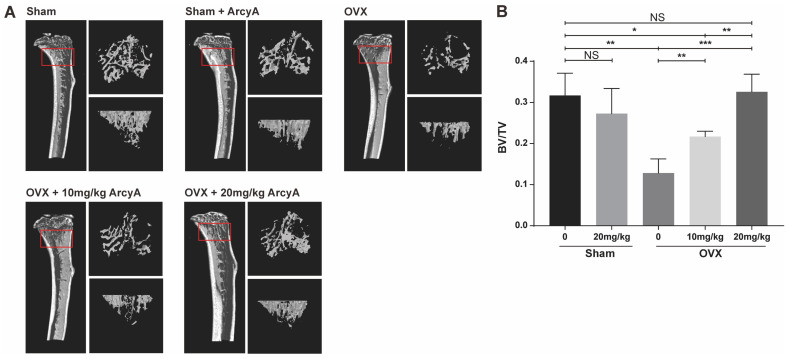
(**A**) 3D high-resolution micro-CT images of the tibia microstructure. Regions measuring 1.8 mm in height, located below the tibial epiphyseal plate, were selected as the regions of interest (ROI) for bone analysis. The approximate locations are highlighted by the red box. On the right side of each tibia figure, two additional views display the trabecular structure within the ROI from two perspectives: one parallel to and one perpendicular to the long axis of the tibia. (**B**) Quantitative analysis of the ratio of bone volume/total volume (BV/TV; volume of mineralized bone per unit volume of the sample) in each group. Data (N = 4) are presented as mean ± SD, with statistical significance indicated as * *p* ≤ 0.05, ** *p* ≤ 0.01, *** *p* ≤ 0.001, ns indicates no significant difference.

Based on the promising in vitro results, in vivo experiments were conducted to determine whether the effect of ArcyA could be replicated and extended in a complex biological system (Figure 6).

The results from the mouse model echo those of our previous molecular tests and TRAP staining. The 3D reconstruction images from CT scans demonstrate that treatment groups (at doses of 10 mg/kg and 20 mg/kg) had more bone mass than the ovariectomy (OVX) non-treatment group. Moreover, bone mass in the high-dose treatment group was comparable to that of the control group, with quantified data of BV/TV providing further clarity.

The BV/TV ratio in the OVX group was lower than that in Sham group, confirming that ovariectomy induced bone loss in the mice. However, there was no significant difference in BV/TV ratio between the sham and the sham + ArcyA groups, ruling out any effect of ArcyA on physiologically normal bone tissue. In the 10 mg/kg treatment group, BV/TV showed a marked increase, which was further enhanced when the dose was increased to 20 mg/kg. Meanwhile, the 20 mg/kg treatment group displayed higher BV/TV (*p* = 0.0028). Furthermore, the bone mass in the 10 mg/kg treatment group remained lower than that in the sham group. In contrast, there was no significant difference between the sham and the 20 mg/kg treatment groups.

## 3. Discussion

To screen potential drugs against OC differentiation, we evaluated candidates from two perspectives: cytotoxicity and TRAP staining. A candidate drug should inhibit OC differentiation and/or OC activity without exhibiting cytotoxicity. Therefore, the CCK-8 assay was performed to evaluate the biosafety of ArcyA, which confirmed the results of TRAP staining.

**Figure 7:**
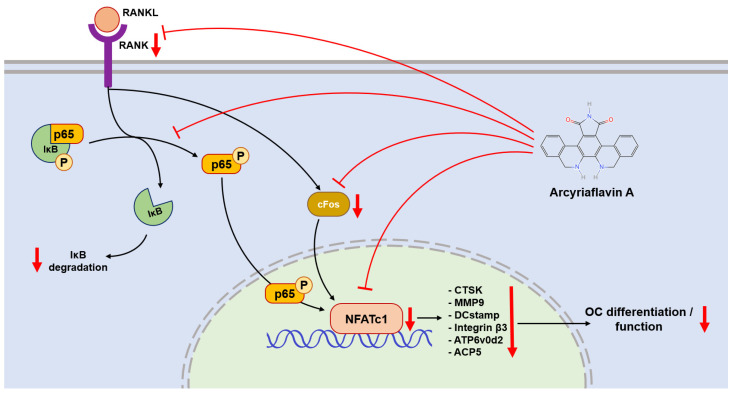
NFATc1 plays a central role in regulating the expression of downstream genes and proteins, including CTSK, MMP9, DCstamp, integrin β3, ATP6v0d2, and ACP5. The NF-κB signaling pathway bridges between RANK-RANKL activation and NFATc1 expression. ArcyA downregulated (arrows pointing down) the gene encoding RANK and inhibited the NF-κB signaling pathway. This was mediated in part by a downregulation of IκB degradation. In addition, ArcyA reduced the expressions of cFos and NFATc1, thereby suppressing the downstream genes involved in OC formation and resorption. The molecular structure was generated using the InDraw software V6 (https://www.integle.com/static/indraw; accessed on 23 February 2025).

In the process of RANKL-induced OC differentiation, nuclear factor of activated T cells, cytoplasmic 1 (NFATc1) plays a central role [21]. RANKL and RANK activate NFATc1, driving it into an auto-amplification phase [22], which allows NFATc1 to continuously stimulate the expression of downstream genes during the late stage of differentiation. This also allows the detection of NFATc1 in the terminal stage of OC formation. In this context, c-Fos is involved, and plays a critical role in both the initial activation and continuous transcription of NFATc1 [23]. Therefore, the gene expression levels of TNFrsf11a (encoding RANK), c-Fos, and NFATc1 were used to evaluate the activation of OC. In our study, the PCR and western blot results showed downregulation after ArcyA administration (Figure 7).

Another link in the RANKL–RANK–NFATc1 axis is the NF-κB signaling pathway. In this pathway, the phosphorylation site on NF-κB is initially blocked by IκB. The binding of RANKL and RANK triggers the phosphorylation of IκB, leading to its dissociation from NF-κB. Dissociation exposes the phosphorylation site on NF-κB, allowing its phosphorylation, nuclear translocation, and the subsequent activation of NFATc1 [24,25]. Therefore, we measured the protein level of IκB at the early stage. Compared with the non-treated group, ArcyA increased IκB levels, demonstrating that ArcyA inhibits the activation of the NF-κB pathway.

Further testing revealed that downstream genes and proteins related to the formation and function of OCs were also downregulated by ArcyA compared to the non-treatment group. DC-stamp and ATP6v0d2 are involved in OC fusion and maturation [26,27]; the reduction in these two genes provides molecular support for the TRAP and fluorescence staining results. CTSK, integrin β3, and MMP9 contribute to the bone resorption function of OCs [28,29,30,31]; the reduction in these genes and proteins supports the resorption assay results. The apatite calcium phosphate coating layer simulates the real bone tissue and external cellular matrix (ECM) to facilitate the measurement of OC resorption ability. The resorption pits in the coating layer visualize the differences between the groups, with the relatively smaller resorption areas attributed to the downregulation of the above-mentioned genes.

To extend our research from animal cells to human cells, the concentration range was further subdivided to accurately assess the inhibitory effect of ArcyA on human PBMC-derived OCs. The results show that a concentration as low as one tenth (0.1 μM) was sufficient to produce statistically significant effects, suggesting that human mononuclear cells are more sensitive to ArcyA than mouse BMMs. However, further in-depth experiments are needed to gain a deeper understanding of the detailed mechanisms in human cells.

Micro-CT technology allows the assessment of bone mass, and provides a comprehensive understanding of the drug’s effect in vivo [32,33]. In our study, trabecular 3D-reconstruction imaging and quantitative analysis showed that OVX mice treated with ArcyA exhibited higher BV/TV than the non-treated group without compromising the health of mice, confirming the alleviating effect of ArcyA on OVX-induced osteoporosis. Furthermore, this effect increased with increasing concentration.

In the future, we plan to expand these studies to a larger cohort and explore additional routes of administration, such as oral and intravenous administration, to identify more effective treatment methods. In addition, we will investigate the detailed effects of the drug on aging animals undergoing long-term treatment.

## 4. Materials and Methods

### 4.1. Primary Mouse Cells Culture

The primary cells used in our experiments were derived from fresh bone marrow, specifically bone marrow-derived macrophages (BMMs). Bone marrow was flushed out from the tibiae and femora of 6-week-old mice using a 1 mL syringe under sterile conditions. The bone marrow cells were cultured with complete α-MEM medium supplied with 50 ng/mL mouse M-CSF (mouse macrophage colony-stimulating factor) (Miltenyi Biotec, Bergisch Gladbach, Germany) in a 100 × 22 mm tissue culture dish (Th. Geyer, Renningen, Germany).

The composition of the complete α-MEM medium was as follows: 500 mL alpha minimum essential medium (α-MEM), 50 mL fetal bovine serum of U.S. origin, 10 mL Penicillin–Streptomycin (P/S), and 10 mL L-Glutamine 200 mM. All reagents above were obtained from Gibco, Thermo Fisher (Waltham, MA, USA). Additionally, 1 g of HEPES (Carl Roth, Karlsruhe, Germany) was added into the system above for pH stabilization.

After 24 h of culture in the presence of M-CSF, adherent cells from the suspended bone marrow were considered BMMs. The BMMs were trypsinized and seeded into 96-well plates or 6-well plates (Greiner, Kremsmünster, Austria) as needed. The transferred BMMs were cultured in complete α-MEM medium in the presence of both mouse M-CSF and mouse RANKL (Miltenyi Biotec, Bergisch Gladbach, Germany) for 5–9 days to be induced into OCs.

### 4.2. Biosafety Assessment of ArcyA

Synthetic ArcyA (CAS number 118458-54-1), a natural polyether compound originally isolated from the marine invertebrate *Eudistoma* sp., was purchased from Santa Cruz Biotechnology (Dallas, TX, USA). A 10 mg ArcyA solid powder was dissolved in 3.07 mL of dimethyl sulfoxide (DMSO, AppliChem GmbH, Darmstadt, Germany) to create a 10 mM stock solution, which was then stored at −20 °C for long-term preservation. The stock solution was further diluted in the medium to achieve the desired working concentrations.

BMMs were transferred from 10 cm plates to 96-well plates at a concentration of 1 × 10^4^ cells per well. The BMMs were cultured in 100 μL complete α-MEM medium, as described above, supplemented with 50 ng/mL M-CSF for a 24 h adaption period. Subsequently, the cells were divided into 3 groups and ArcyA was added to each group at concentrations of 0 μM, 1 μM, and 5 μM. The cells were cultured for 5 days with medium changes every two days. On day 5, 10 μL of WST-8 solution (Cell Counting Kit-8; Dojindo Laboratories, Kumamoto, Japan) was applied to 24 wells and incubated at 37 °C for 4 h. Every group included one blank control well containing 100 μL medium without cells. After incubation, the absorbance of 450 nm was measured by the SPARK^®^ Microplate reader (Tecan Deutschland Gmbh, Crailsheim, Germany).

### 4.3. TRAP and Fluorescence Staining

BMMs were transferred and seeded into 96-well plates at a density of 1 × 10^4^ cells per well. In the control groups, BMMs were cultured with complex α-MEM medium containing 50 ng/mL M-CSF only, while the positive control group received 50 ng/mL M-CSF and 50 ng/mL RANKL. The two treatment groups were supplemented with 50 ng/mL M-CSF, 50 ng/mL RANKL, and either 1 μM or 5 μM ArcyA separately. The cells were cultured for 5 days with medium changes every two days. The volume of medium was 200 μL per well.

After culturing, the cells were washed with PBS, then fixed with 4% formalin (Roti^®^Histofix, Carl Roth, Karlsruhe, Germany) for TRAP staining, and 4% paraformaldehyde (MORPHISTO GmbH, Offenbach, Germany) for fluorescent staining.

For TRAP staining, fixed cells were washed with PBS three times and incubated in TRAP buffer at room temperature for 10 min, followed by incubation in TRAP dye solution at 37 °C for 1 h. The TRAP buffer was prepared using sodium acetate (AppliChem GmbH, Gatersleben, Germany) and sodium tartrate dihydrate (Carl Roth, Karlsruhe, Germany). Naphthol AS-Mix Phosphate (AppliChem GmbH, Gatersleben, Germany), N-N-Dimethylformamide and Fast Red Violet LB Salt (Sigma-Aldrich, St. Louis, MO, USA) were dissolved in TRAP buffer to prepare the TRAP dye solution.

The staining results were photographed and analyzed under a bright-field microscope. Red-stained cells with more than three nuclei were classified as OCs. Furthermore, five identical areas (0.01 mm^2^) were selected at the center and periphery of each well as sampling regions, and then the numbers of TRAP-positive cells were counted.

For fluorescent staining, fixed cells were washed with PBS three times and treated with 0.1% Triton X-100 (Sigma-Aldrich, St. Louis, MO, USA) to increase membrane permeability. Cells were then incubated in 1% bovine serum albumin (BSA, Carl Roth, Karlsruhe, Germany) for 30 min. Sequential fluorescent staining was performed on the cytoskeleton and nucleus using Phalloidin-iFluor 647 and DAPI (Abcam, Cambridge, UK), respectively. The staining results were photographed and analyzed under a fluorescent microscope. Additionally, the number of OC nuclei in the sampling regions was counted, and the proportion of multinucleated cells was calculated.

The bright field and fluorescent microscope and microimaging system comprised an Olympus IX81 microscope equipped with a DP80 microphotographic camera (Olympus Corporation, Tokyo, Japan).

### 4.4. Resorption Assay

Based on a protocol by Tas and Bhaduri [34], 96-well plates were coated with bone-like apatite calcium phosphate. BMMs were seeded onto the coated 96-well plate at a density of 1 × 10^4^ cells per well. The group settings were the same as described in the “TRAP and Fluorescence staining” section. The cells were cultured for 7 days, with medium changes every two days. The volume of medium was 200 μL per well.

After the culturing period, all cells were removed from each well using 5% sodium thiosulfate. The plate was then incubated with silver nitrate solution in the dark for 30 min, during which the residual coating was stained black by silver nitrate. Subsequently, the stained coating was neutralized and fixed using sodium carbonate–formaldehyde solution. All the above chemical reagents were obtained from Carl Roth (Karlsruhe, Germany). Finally, a small amount of pure ethanol was added to the wells for long-term preservation.

During the process of OC differentiation, the OCs resorbed the apatite calcium phosphate coating. As a result, resorption pits appeared transparent under microscope, and were easily distinguishable from the remaining black-stained coating. ImageJ software (Ver. 1.46r) was applied to calculate the aeras of resorption pits.

### 4.5. Real-Time Quantitative Polymerase Chain Reaction

BMMs were seeded in 6-well plates at a density of 3.0 × 10^5^ cells per well and cultured in the aforementioned complete α-MEM medium, with the medium being changed every two days. The volume of medium was 2 mL per well.

For the negative control (NC) group, the medium contained 50 ng/mL M-CSF; for the positive control (PC) group, 50 ng/mL M-CSF and 50 ng/mL RANKL; for the two treatment groups, 50 ng/mL M-CSF, 50 ng/mL RANKL, and 1 μM or 5 μM ArcyA separately. After 5 days of culture, cells were harvested using TRIzol^®^ Reagent (Invitrogen, Thermo Fisher, Waltham, MA, USA). Total RNA was extracted by the TRIzol-Chloroform method with chloroform (AppliChem GmbH, Darmstadt, Germany) applied for RNA phase separation, 2-Propanol (AppliChem GmbH, Darmstadt, Germany) for RNA precipitation, and 75% ethanol (AppliChem GmbH, Darmstadt, Germany) for RNA purification. RNA concentrations were measured using NanoPhotometer^®^ N60 (Implen GmbH, München, Germany), and subsequently reverse-transcribed into single-stranded cDNA with a High-Capacity cDNA Reverse Transcription Kit (Applied Biosystems, Thermo Fisher, Waltham, MA, USA). Target genes’ expression levels were quantified with PowerUp™ SYBR™ Green Master Mix (Applied Biosystems, Thermo Fisher, Waltham, MA, USA) on the Light-Cycler^®^ 480II System (Roche, Basel, Switzerland). GAPDH was used as normalization reference, and the relative expression levels were analyzed in 2^−ΔΔCq^ method. The primer sequences are listed in Table 1.

### 4.6. Western Blot Analysis

To analyze function- and differentiation-related proteins, 3.0 × 10^5^ BMMs were seeded into 6-well plates and cultured in the aforementioned complete α-MEM medium for 5 days. The volume of medium was 2 mL per well.

For the negative control (NC) group, the medium contained 50 ng/mL M-CSF; for the positive control (PC) group, 50 ng/mL M-CSF and 50 ng/mL RANKL; for the treatment groups, 50 ng/mL M-CSF, 50 ng/mL RANKL, and 5 μM ArcyA. After 5 days of culture, RIPA buffer (Carl Roth, Karlsruhe, Germany) was used to collect total protein from each group.

For the signaling pathway protein, 1.0 × 10^6^ BMMs were seeded into 6-well plates, and cultured in α-MEM medium without FBS and RANKL for 12 h to starve the cells. Next, 5 μM ArcyA was added to the treatment groups, while an equal volume of PBS was added to the control groups. The BMMs were cultured for another 2 h to allow for ArcyA uptake. Subsequently, the old medium was replaced with medium containing 50 ng/mL RANKL. Protein samples were collected using RIPA after RANKL induction at 10 min, 20 min, and 60 min. The sample collected from the group without RANKL induction was designated as the control group (0 min group).

First, protein samples were centrifuged at 4680 rpm for 5 min to remove nuclear acid. Next, the protein concentration of each sample was determined using the Pierce™ BCA Protein assay kit (Thermo Fisher, Waltham, MA, USA), and all samples were adjusted to the same concentration level.

Standardized samples were mixed with loading buffer at a ratio of 3:1; 1 mL loading buffer was prepared from Laemmli sample buffer (Bio-Rad, Berkeley, CA, USA) and 2-Mercaptoethanol (AppliChem GmbH, Darmstadt, Germany). The samples in the loading buffer were boiled at 95 °C for 5 min to denature the proteins and eliminate their secondary structure. The boiled samples were loaded onto sodium dodecyl sulfate-polyacrylamide gel (10%) for electrophoresis to separate the total proteins based on their molecular weight.

The separated protein samples were transferred onto nitrocellulose membranes using the Trans-Blot^®^ Turbo™ transfer system (Bio-Rad, Berkeley, CA, USA), and blocked with 5% BSA (Carl Roth, Karlsruhe, Germany) for 1 h. The blocked membranes were then incubated with the following primary antibodies: anti-NFATc1 (1:1000); anti-Integrin beta3 (1:1000); anti-IkB (L35A5, 1:1000); anti-Tubulin (D3U1W, 1:5000); and anti-GAPDH (D16H11, 1:5000) produced by CST (Cell Signaling Technology, Danvers, MA, USA), and anti-cFos (T.142.5, 1:1000) and anti-CTSK (Cathepsin K Polyclonal Antibody, 1:1000) produced by Invitrogen (Thermo Fisher, Waltham, MA, USA).

### 4.7. Assessment of Drug-Induced Inhibition of OC Differentiation

The protocol of human OC differentiation used in this study was adapted from the method reported by Brom et al. (2023) [35]. Human whole blood samples were collected from five donors using EDTA-monovettes. The collected blood was then diluted with PBS at a 1:1 ratio. Peripheral blood mononuclear cells (PBMCs) were isolated using SepMate™-50 tubes (STEMCELL technologies, Vancouver, BC, Canada). Here, 15 mL of BioColl^®^ separation solution (BIO&SELL, Feucht, Germany) and diluted blood sample were sequentially added into SepMate™ tubes. The tubes were centrifuged at 2800 rpm for 10 min at room temperature. The top layer containing the PBMCs was transferred to a new tube.

PBMCs were seeded in a 12-well ibidi chamber (Ibidi GmbH, Gräfelfing, Germany) at a density of 5.0 × 10^5^ cells per well. In each group, PBMCs were cultured in the complete α-MEM medium supplemented with 50 ng/mL human M-CSF, 50 ng/mL human RANKL (Miltenyi Biotec, Bergisch Gladbach, Germany), and 10 nM Vitamin D (Sigma-Aldrich, St. Louis, MO, USA) for human OC differentiation. ArcyA was added to the treatment groups in varying amounts to reach final concentrations of 0.1 μM, 1 μM, 2.5 μM, and 5 μM. Additionally, 8 µL DMSO was added to the DMSO control group, while an equivalent volume of PBS at the same volume was added to the control group. The cells were cultured for 12 days, with medium changes every two days. The volume of medium was 200 μL per well.

After culturing, the cells were washed with PBS, then fixed with 4% formalin. TRAP staining and cell counting methods were performed as described in the section on “TRAP and fluorescence staining”.

### 4.8. Ovariectomy Mouse Model and Micro-CT Scanning

All animal experiments were approved by the Ethics Committee of the Second Hospital of Shanxi Medical University (approval No. DW2023028). Female 10-week-old C57BL/6 mice were obtained from the Animal Center of Shanxi Medical University in China (n = 20) and randomly separated into five groups, as follows: sham group (n = 4), sham + ArcyA group (n = 4), OVX group (n = 4), OVX + 10 mg/kg ArcyA group (n = 4), and OVX + 20 mg/kg ArcyA group (n = 4).

All mice received general anesthesia by inhaled isoflurane (RWD Life Science, Shenzhen, China). After preoperative preparation and skin disinfection, bilateral ovarian ligation and resection were performed via dorsal approach. Mice in the sham group and Sham + ArcyA group underwent a sham operation.

Following surgery, all mice were placed in a dark, quiet, and warm cage to minimize environmental stimuli and reduce unnecessary stress during anesthesia recovery. The mice were housed together within their respective groups and allowed to adapt for about one week until the wounds had healed. Afterwards, intraperitoneal drug injections were administered at a dosage of 10 mg/kg for OVX + 10 mg/kg ArcyA group, 20 mg/kg for sham + ArcyA and OVX + 20 mg/kg ArcyA groups. The sham and OVX groups received normal saline. The interventions were administrated once every five days for a total duration of eight weeks.

After the eight-week treatment, the mice were euthanized. One tibia from each mouse was extracted and fixed in 4% paraformaldehyde (Leagene, Beijing, China) for 24 h before micro-CT scanning. The tibia samples were scanned under the BRUKER skyscan1176 μCT instrument (Bruker Daltonic Inc., Billerica, MA, USA) with the following setup: 50 kV scanning voltage, 500 μA scanning current, 9 μm spatial resolution, and 1600 × 2672-pixel image matrix. The regions of interest (ROI) for bone analysis were located below the tibia epiphyseal plate with a scanning height of approximately 1.8 mm. NRecon reconstruction software (Ver. 2.0) and CTAn (CT-Analyzer, Ver. 1.18) were applied for three-dimensional image rebuilding and quantitative analysis. BV/TV was the primary parameter of interest. The MicroCT scanning procedure and parameters were assessed in accordance with the guidelines provided by Center for Advanced Orthopedic Studies [33].

### 4.9. Statistical Analysis

All quantitative data are presented as the mean ± standard deviation. Statistical analyses were conducted using one-way analysis of variance (ANOVA) followed by Tukey’s post hoc test with GraphPad Prism 8. Compared to the control, all data are presented as the means ± SDs; * *p* ≤ 0.05, ** *p* ≤ 0.01, *** *p* ≤ 0.001, **** *p* ≤ 0.0001.

## 5. Conclusions

The aim of this study was to investigate the inhibitory effect of ArcyA on OC differentiation and function in the context of osteoporosis. Our results highlight the potential use of ArcyA as a novel therapeutic agent for osteoporosis from multiple perspectives, including molecular and cellular biological assays as well as in vivo animal models.

Specifically, ArcyA exerted a significant inhibitory effect on OC differentiation and bone resorption activity, and it downregulated corresponding marker genes and proteins. In addition, the biosafety and ability to reduce bone loss were supported by our in vivo results. Together with the applicability of ArcyA in human cell models, the translational potential of this compound to be developed as a clinically relevant treatment was further strengthened.

In conclusion, this study has provided some compelling evidence that ArcyA may serve as a promising candidate for the treatment of osteoporosis. Future investigations are needed to better understand the underlying molecular mechanisms of ArcyA and to pave the way for clinical trials, including dose optimization, long-term efficacy and safety evaluation. In addition, exploring potential synergies between ArcyA and other osteoporosis treatments may offer new ways to improve patient outcomes.

## Figures and Tables

**Table 1:** Primer sequences used in qRT-PCR.

Gene	Froward (5′-3′)	Reverse (5′-3′)	Tm (°C)
GAPDH	AGG TCG GTG TGA ACG GAT TTG	TGT AGA CCA TGT AGT TGA GGT	60
NFATc1	GGT GCC TTT TGC GAG CAG TAT C	CGT ATG GAC CAG AAT GTG ACG G	60
c-Fos	GGG AAT GGT GAA GAC CGT GTC A	GCA GCC ATC TTA TTC CGT TCC C	60
TNFrsf11a	GGA CAA CGG AAT CAG ATG TGG TC	CCA CAG AGA TGA AGA GGA GCA G	60
CTSK	CCA GTG GGA GCT ATG GAA GA	AAG TGG TTC ATG GCC AGT TC	60
MMP9	GCT GAC TAC GAT AAG GAC GGC A	TAG TGG TGC AGG CAG AGT AGG A	60
DC-stamp	TTT GCC GCT GTG GAC TAT CTG C	GCA GAA TCA TGG ACG ACT CCT TG	60
ACP5	CAG CAG CCA AGG AGG ACT AC	ACA TAG CCC ACA CCG TTC TC	59
Integrin β3	GTG AGT GCG ATG ACT TCT CCT G	CAG GTG TCA GTG CGT GTA GTA C	60
ATP6v0d2	ACG GTG ATG TCA CAG CAG ACG T	CTC TGG ATA GAG CCT GCC GCA	60

## Data Availability

The original contributions presented in the study are included in the article, and further inquiries can be directed to the corresponding author.
